# Supplementary material for: Synergistic information supports modality integration and flexible learning in neural networks solving multiple tasks
Source: PLoS Comput Biol. 2024 Jun 3;20(6):e1012178. doi: 10.1371/journal.pcbi.1012178 (PMC11175422; doi:10.1371/journal.pcbi.1012178)
Supplement: S1 Text — Full-order and 2nd-order measures exhibit similar behavior for the COPY task. Values represent individual data points, and means ± SEM. Fig B in S1 Text. Full-order and 2nd-order measures exhibit similar behavior for the XOR task. Values represent individual data points, and means ± SEM. Fig C in S1 Text. Networks with different layer sizes (ten versus twenty neurons) exhibit similar behavior for the COPY task (2nd-order). Values represent individual data points, and means ± SEM. Fig D in S1 Text. Networks with different layer sizes (ten versus twenty neurons) exhibit similar behavior for the XOR task (2nd-order). Values represent individual data points, and means ± SEM. Fig E in S1 Text. Effects of lesions and dropout on network information profiles replicate in larger networks (twenty neurons rather than ten). (a) (****P<0.0001, independent samples t test; n = 20). Values represent probability density functions. (b) (*P<0.05, **P<0.01, ***P<0.001, ****P<0.0001, paired samples t test with Benjamini-Hochberg False Discovery rate correction; n = 20). Values represent means ± SEM. Fig F in S1 Text. Dropout removes irrelevant redundant and synergistic information about the input in the COPY task, but not the XOR task (*P<0.05, **P<0.01, independent samples t test; n = 20). Values represent probability density functions. Fig G in S1 Text. Relation of compositional tasks and synergy in Animal-AI using pairwise raycast-position sources. (a) (**P<0.01, paired samples t test; n = 20). Values represent probability density functions. (b) (n.s., not significant, paired samples t test; n = 20). Values represent probability density functions. (c) Values represent probability density functions. Fig H in S1 Text. Layer-wise comparison for COPY task with different levels of dropout applied (*P<0.05, **P<0.01, ***P<0.001, ****P<0.0001, independent samples t test; n = 20). Values represent probability density functions. Fig I in S1 Text. Layer-wise comparison for XOR task with differ [file pcbi.1012178.s001.docx]

Supporting Information for

**Synergistic information supports modality integration and flexible learning in neural networks solving multiple tasks**

Alexandra M. Proca, Fernando E. Rosas, Andrea I. Luppi, Daniel Bor, Matthew Crosby,

Pedro A.M. Mediano

**This file includes:**

Figs A to X

Table A

Fig A. Full-order and 2^nd^-order measures exhibit similar behavior for the COPY task. Values represent individual data points, and means ± SEM.

Fig B. Full-order and 2^nd^-order measures exhibit similar behavior for the XOR task. Values represent individual data points, and means ± SEM.

Fig C. Networks with different layer sizes (ten versus twenty neurons) exhibit similar behavior for the COPY task (2^nd^-order). Values represent individual data points, and means ± SEM.

Fig D. Networks with different layer sizes (ten versus twenty neurons) exhibit similar behavior for the XOR task (2^nd^-order). Values represent individual data points, and means ± SEM.

Fig E. Effects of lesions and dropout on network information profiles replicate in larger networks (twenty neurons rather than ten). (a) (^****^*P*<0.0001, independent samples *t* test; *n*=20). Values represent probability density functions. (b) (^*^*P*<0.05, ^**^*P*<0.01, ^***^*P*<0.001, ^****^*P*<0.0001, paired samples *t* test with Benjamini-Hochberg False Discovery rate correction; *n*=20). Values represent means ± SEM.

Fig F. Dropout removes irrelevant redundant and synergistic information about the input in the COPY task, but not the XOR task (^*^*P*<0.05, ^**^*P*<0.01, independent samples *t* test; *n*=20). Values represent probability density functions.

Fig G. Relation of compositional tasks and synergy in Animal-AI using pairwise raycast-position sources. (a) (^**^*P*<0.01, paired samples *t* test; *n*=20). Values represent probability density functions. (b) (n.s., not significant, paired samples *t* test; *n*=20). Values represent probability density functions. (c) Values represent probability density functions.

Fig H. Layer-wise comparison for COPY task with different levels of dropout applied (^*^*P*<0.05, ^**^*P*<0.01, ^***^*P*<0.001, ^****^*P*<0.0001, independent samples *t* test; *n*=20). Values represent probability density functions.

Fig I. Layer-wise comparison for XOR task with different levels of dropout applied (^*^*P*<0.05, ^**^*P*<0.01, ^****^*P*<0.0001, independent samples *t* test; *n*=20). Values represent probability density functions.

Fig J. Layer-wise comparison at the end of training for Animal-AI tasks using pairwise raycast sources (^**^*P*<0.01, ^****^*P*<0.0001, independent samples *t* test; *n*=20). Values represent probability density functions.

Fig K. Layer-wise comparison at the end of training for Animal-AI tasks using pairwise raycast-position sources (^**^*P*<0.01, ^****^*P*<0.0001, independent samples *t* test; *n*=20). Values represent probability density functions.

**Figures replicated using** $\boldsymbol{I}_{\text{min}}$ **redundancy function**

Fig L. Full-order and 2^nd^-order measures exhibit similar behavior for the COPY task replicated using $\boldsymbol{I}_{\text{min}}$ redundancy function. Values represent individual data points, and means ± SEM.

Fig M. Full-order and 2^nd^-order measures exhibit similar behavior for the XOR task replicated using $\boldsymbol{I}_{\text{min}}$ redundancy function. Values represent individual data points, and means ± SEM.

Fig N. Networks with different hidden layer sizes (ten versus twenty neurons) exhibit similar behavior for the COPY task (2^nd^-order) replicated using $\boldsymbol{I}_{\text{min}}$ redundancy function. Values represent individual data points, and means ± SEM.

Fig O. Networks with different hidden layer sizes (ten versus twenty neurons) exhibit similar behavior for the XOR task (2^nd^-order) replicated using $\boldsymbol{I}_{\text{min}}$ redundancy function. Values represent individual data points, and means ± SEM.

Fig P. Dropout removes irrelevant redundant and synergistic information about the input in the COPY task, but not the XOR task replicated using $\boldsymbol{I}_{\text{min}}$ redundancy function (^*^*P*<0.05, ^**^*P*<0.01, independent samples *t* test; *n*=20). Values represent probability density functions.

Fig Q. Effects of lesions and dropout on network information profiles replicated using $\boldsymbol{I}_{\text{min}}$ redundancy function. (a) (^*^*P*<0.05, ^***^*P*<0.001, ^****^*P*<0.0001, independent samples *t* test; *n*=20). Values represent probability density functions. (b) (^*^*P*<0.05, ^**^*P*<0.01, paired samples *t* test with Benjamini-Hochberg False Discovery rate correction; *n*=20). Values represent means ± SEM.

Fig R. Effects of lesions and dropout on network information profiles replicate in larger networks (twenty neurons rather than ten) replicated using $\boldsymbol{I}_{\text{min}}$ redundancy function. (a) (^****^*P*<0.0001, independent samples *t* test; *n*=20). Values represent probability density functions. (b) (^*^*P*<0.05, ^**^*P*<0.01, ^***^*P*<0.001, ^****^*P*<0.0001, paired samples *t* test with Benjamini-Hochberg False Discovery rate correction; *n*=20). Values represent means ± SEM.

Fig S. Relation of compositional tasks and synergy in Animal-AI using pairwise raycast sources replicated using $\boldsymbol{I}_{\text{min}}$ redundancy function. (a) (^**^*P*<0.01, paired samples *t* test; *n*=20). Values represent probability density functions. (b) (n.s., not significant, paired samples *t* test; *n*=20. Values represent probability density functions. (c) Distance XOR refers to Distance 10 XOR. Values represent probability density functions.

Fig T. Relation of compositional tasks and synergy in Animal-AI using pairwise raycast-position sources replicated using $\boldsymbol{I}_{\text{min}}$ redundancy function. (a) (^**^*P*<0.01, paired samples *t* test; *n*=20). Values represent probability density functions. (b) (n.s., not significant, paired samples *t* test; *n*=20. Values represent probability density functions. (c) Distance XOR refers to Distance 10 XOR. Values represent probability density functions.

Fig U. Layer-wise comparison for COPY task with different levels of dropout applied replicated using $\boldsymbol{I}_{\text{min}}$ redundancy function (^**^*P*<0.01, ^***^*P*<0.001, ^****^*P*<0.0001, independent samples *t* test; *n*=20). Values represent probability density functions.

Fig V. Layer-wise comparison for XOR task with different levels of dropout applied replicated using $\boldsymbol{I}_{\text{min}}$ redundancy function (^****^*P*<0.0001, independent samples *t* test; *n*=20). Values represent probability density functions.

Fig W. Layer-wise comparison at the end of training for Animal-AI tasks using pairwise raycast sources replicated using $\boldsymbol{I}_{\text{min}}$ redundancy function (^*^*P*<0.05, ^****^*P*<0.0001, independent samples *t* test; *n*=20). Values represent probability density functions.

Fig X. Layer-wise comparison at the end of training for Animal-AI tasks using pairwise raycast-position sources replicated using $\boldsymbol{I}_{\text{min}}$ redundancy function (^*^*P*<0.05, ^***^*P*<0.001, ^****^*P*<0.0001, independent samples *t* test; *n*=20). Values represent probability density functions.

|  |  | Original |  | IQR |  |  |  |  |  |  |  |
| --- | --- | --- | --- | --- | --- | --- | --- | --- | --- | --- | --- |
|  |  | 3 bins, max 5 |  | 3 bins |  | 4 bins |  | 5 bins |  | 10 bins |  |
|  |  | $I_{\text{MMI}}$ | $I_{\text{min}}$ | $I_{\text{MMI}}$ | $I_{\text{min}}$ | $I_{\text{MMI}}$ | $I_{\text{min}}$ | $I_{\text{MMI}}$ | $I_{\text{min}}$ | $I_{\text{MMI}}$ | $I_{\text{min}}$ |
| Logic gate experiments | Difference in redundancy of input for dropout $p=0.0,0.5$ in COPY task | 0.16 (^*^*P*<0.05) | 0.16 (^*^*P*<0.05) | 0.13 (^*^*P*<0.05) | 0.13 (^*^*P*<0.05) | 0.13 (^*^*P*<0.05) | 0.13 (^*^*P*<0.05) | 0.12 (^***^*P*<0.001) | 0.12 (^***^*P*<0.001) | 0.12 (^***^*P*<0.001) | 0.12 (^***^*P*<0.001) |
|  | Difference in synergy of input for dropout $p=0.0,0.5$ in COPY task | 0.24 (^**^*P*<0.01) | 0.24 (^**^*P*<0.01) | 0.13 (^*^*P*<0.05) | 0.13 (^*^*P*<0.05) | 0.13 (^*^*P*<0.05) | 0.13 (^*^*P*<0.05) | 0.12 (^***^*P*<0.001) | 0.12 (^***^*P*<0.001) | 0.12 (^***^*P*<0.001) | 0.12 (^***^*P*<0.001) |
|  | Difference in redundancy of input for dropout $p=0.0,0.5$ in XOR task | -0.02 (n.s.) | -0.02 (n.s.) | 0 (n.s.) | 0 (n.s.) | 0 (n.s.) | 0 (n.s.) | 0 (n.s.) | 0 (n.s.) | 0 (n.s.) | 0 (n.s.) |
|  | Difference in synergy of input for dropout $p=0.0,0.5$ in XOR task | -0.02 (n.s.) | -0.02 (n.s.) | 0 (n.s.) | 0 (n.s.) | 0 (n.s.) | 0 (n.s.) | 0 (n.s.) | 0 (n.s.) | 0 (n.s.) | 0 (n.s.) |
|  | Difference in redundancy of layer 1 for dropout $p=0.0,0.5$ in COPY task | 0.4 (^****^*P*<0.0001) | 0.41 (^****^*P*<0.0001) | 0.27 (^**^*P*<0.01) | 0.29 (^***^*P*<0.001) | 0.19 (^*^*P*<0.05) | 0.21 (^*^*P*<0.05) | 0.15 (n.s.) | 0.15 (n.s.) | 0.07 (n.s.) | 0.07 (n.s.) |
|  | Difference in redundancy of layer 2 for dropout $p=0.0,0.5$ in COPY task | 0.6 (^****^*P*<0.0001) | 0.6 (^****^*P*<0.0001) | 0.63 (^****^*P*<0.0001) | 0.63 (^****^*P*<0.0001) | 0.58 (^****^*P*<0.0001) | 0.58 (^****^*P*<0.0001) | 0.59 (^****^*P*<0.0001) | 0.59 (^****^*P*<0.0001) | 0.59 (^****^*P*<0.0001) | 0.59 (^****^*P*<0.0001) |
|  | Difference in redundancy of layer 1 for dropout $p=0.0,0.5$ in XOR task | 0.08 (n.s.) | 0.07 (^*^*P*<0.05) | 0.04 (n.s.) | 0.01 (n.s.) | 0.01 (n.s.) | -0.02 (n.s.) | 0.0001 (n.s.) | -0.03 (n.s.) | -0.03 (n.s.) | -0.05 (n.s.) |
|  | Difference in redundancy of layer 2 for dropout $p=0.0,0.5$ in XOR task | 0.35 (^***^*P*<0.001) | 0.35 (^***^*P*<0.001) | 0.29 (^**^*P*<0.01) | 0.28 (^**^*P*<0.01) | 0.27 (^**^*P*<0.01) | 0.26 (^**^*P*<0.01) | 0.26 (^**^*P*<0.01) | 0.25 (^**^*P*<0.01) | 0.21 (^*^*P*<0.05) | 0.21 (^*^*P*<0.05) |
|  | Difference in accuracy between lesioning 6 of the minimally synergistic neurons at dropout $p=0.0,0.5$ in COPY task | 0.2 (^*^*P*<0.05) | 0.03 (n.s.) | 0.15 (n.s.) | 0.05 (n.s.) | 0.28 (^**^*P*<0.01) | 0.15 (n.s.) | 0.23 (^*^*P*<0.05) | 0.28 (^**^*P*<0.01) | 0.25 (^**^*P*<0.01) | 0.2 (^*^*P*<0.05) |
|  | Difference in accuracy between lesioning 6 of the maximally and minimally synergistic neurons at dropout $p=0.5$ in XOR task | 0.35 (^***^*P*<0.001) | 0.23 (^*^*P*<0.05) | 0.25 (^*^*P*<0.05) | 0.18 (^*^*P*<0.05) | 0.23 (^*^*P*<0.05) | 0.2 (^*^*P*<0.05) | 0.23 (^*^*P*<0.05) | 0.2 (^*^*P*<0.05) | 0.2 (^*^*P*<0.05) | 0.13 (n.s.) |
| Animal-AI experiments | Difference in synergy of pairwise-raycast inputs between task 1 and task 2 in 2-bit to 3-bit XOR task | -0.02 (n.s.) | -0.001 (n.s.) | 0.01 (n.s.) | 0.02 (n.s.) | 0.03 (n.s.) | 0.03 (n.s.) | -0.01 (n.s.) | 0.01 (n.s.) | -0.02 (n.s.) | 0.006 (n.s.) |
|  | Difference in synergy of position-raycast inputs between task 1 and task 2 in 2-bit to 3-bit XOR task | 0.04 (n.s.) | 0.02 (n.s.) | 0.04 (n.s.) | 0.02 (n.s.) | 0.004 (n.s.) | 0.001 (n.s.) | 0.05 (n.s.) | 0.03 (n.s.) | 0.04 (^*^*P*<0.05) | -0.003 (n.s.) |
|  | Difference in synergy of layer 1 between task 1 and task 2 in 2-bit to 3-bit XOR task | 0.05 (^**^*P*<0.01) | 0.04 (^**^*P*<0.01) | 0.05 (n.s.) | 0.03 (^*^*P*<0.05) | 0.04 (n.s.) | 0.04 (^*^*P*<0.05) | 0.04 (n.s.) | 0.03 (n.s.) | 0.03 (n.s.) | 0.02 (n.s.) |
|  | Difference in synergy of layer 2 between task 1 and task 2 in  2-bit to 3-bit XOR task | 0.11 (^**^*P*<0.01) | 0.07 (^**^*P*<0.01) | 0.09 (^***^*P*<0.001) | 0.06 (^***^*P*<0.001) | 0.08 (^**^*P*<0.01) | 0.05 (^**^*P*<0.01) | 0.09 (^**^*P*<0.01) | 0.07 (^**^*P*<0.01) | 0.08 (^**^*P*<0.01) | 0.06 (^**^*P*<0.01) |
|  | Difference in synergy of pairwise-raycast inputs between task 1 and task 4 in Distance XOR task | -0.02 (n.s.) | -0.002 (n.s.) | -0.003 (n.s.) | 0.01 (n.s.) | -0.01 (n.s.) | 0.0002 (n.s.) | -0.01 (n.s.) | 0.0001 (n.s.) | -0.01 (n.s.) | 0.009 (n.s.) |
|  | Difference in synergy of position-raycast inputs between task 1 and task 4 in Distance XOR task | -0.01 (n.s.) | -0.02 (n.s.) | 0.02 (n.s.) | 0.01 (n.s.) | -0.003 (n.s.) | 0.01 (n.s.) | 0.02 (n.s.) | 0.002 (n.s.) | 0.008 (n.s.) | -0.003 (n.s.) |
|  | Difference in synergy of layer 1 between task 1 and task 4 in Distance XOR task | -0.01 (n.s.) | -0.003 (n.s.) | 0.002 (n.s.) | 0.01 (n.s.) | 0.001 (n.s.) | 0.01 (n.s.) | -0.001 (n.s.) | 0.01 (n.s.) | -0.01 (n.s.) | -0.01 (n.s.) |
|  | Difference in synergy of layer 2 between task 1 and task 4 in Distance XOR task | 0.02 (n.s.) | -0.02 (n.s.) | 0.02 (n.s.) | 0.01 (n.s.) | 0.02 (n.s.) | 0.005 (n.s.) | 0.02 (n.s.) | 0.01 (n.s.) | 0.01 (n.s.) | 0.002 (n.s.) |

**Table A.** **Summary statistics for logic gate and Animal AI experiments across different binning strategies for discretization (^*^*P*<0.05, ^**^*P*<0.01, ^***^*P*<0.001, ^****^*P*<0.0001, n.s., not significant).** P-values are not corrected for multiple comparisons.
