# Supplementary material for: Synergistic information supports modality integration and flexible learning in neural networks solving multiple tasks
Source: PLoS Comput Biol. 2024 Jun 3;20(6):e1012178. doi: 10.1371/journal.pcbi.1012178 (PMC11175422; doi:10.1371/journal.pcbi.1012178)
Supplement: Attachment — Submitted filename: Comments to the authors.pdf [file pcbi.1012178.s002.pdf]

**Comments to the authors**

This article addresses important aspects of studying artificial and biological neural systems. In particular, it hypothesizes and tests several functions that can be assigned to the individual components of Partial Information Decomposition (PID): it assigns the function of robustness for redundant information, specialization for unique information, and modality interpretation, flexibility as well as efficient coding for synergistic information.
I enjoyed reading the article, learned something new (about PID for instance), and acquired certainty for things already suspected (regarding the effects of dropout or the effects of sequential-vs.-interleaved training for different sets of tasks, for instance). I think that the paper is sound, that it represents a significant contribution, and that it is of interest to the cognitive neuroscience community.
Since I did not find any major content-related or methodological problems, I only have a list of comments and remarks that might rather reflect my own preferences as a reader than actual weaknesses. I don't expect my points to be met in any specific way, but I think that focusing on some of them could improve the paper if you decide to do so:

1. You address the cognitive neuroscience community, which I find appropriate and promising, as I see the main motivation for PID there (more than, e.g., in the purely theoretical field of explainable AI). The relation to cognitive neuroscience is made explicit in three ways: 1., ANNs are somewhat similar to biological neural networks, therefore---although biologically implausible---they can help to extract the fundamental principles of cognitive systems; 2., the 3rd experiment uses the NeuroGym task suit of Molano-Mazon et al. (2022) which has been used for some experiments in decision-making in cognitive neuroscience; and 3., you frequently relate your findings to the information integration in cortical association areas which is largely based on the Luppi et al. (2022) paper.
   Since the third relation is mostly conceptual and not a exactly an outcome of this studies own results, I would find it more interesting to discuss the relations of 1. and 2. more extensively. Relation 1. has been discussed extensively under the name of distributed vs. localist processing for example (see Bowers, 2017, e.g. and referencing earlier work in Parallel distributed Processing); and for relation 2. it might be interesting to discuss experimental results that have used the NeuroGym test suit for animal experiments.
2. Sometimes I thought that the discussions about the failures and the results, especially for the first experiment (e.g. lines 165 to 224), could be made more concise.
3. The redundancy functions $I_{MMI}$ (Barrett, 2015) and $I_{min}$ (William & Beer, 2010) were previously unknown to me, but since this is where the "juice" of PID is, I expected a little intuition as to what both calculations are and also what their functional difference is (either in the background section or in the methods section next where you present the explicit formulas for calculating the measures).
4. With regard to Fig.2 c), I would advice to write that p=0.0 and p=0.5 denote dropout values. It can be deduced from the context (particularly if the image is positioned within the text), but mentioning it in the description as well would make it easier.
5. Maybe it is interesting to mention how this dropout interpretation in terms of redundancy compares to the traditional interpretation of Hinton et al. 2012, Srivastava et al. 2014 as preventing complex co-adaptations.
6. Several ArXiv Preprints seem not to be cited correctly, only the first author and the url is mentioned.

7. Line 436ff: "Synergistic information, however, is also more vulnerable to noise (10), because a disruption in a single source could disrupt information synergistically held together with other sources."
   I am unsure, whether I would call the susceptibility to noise, a "disruption", i.e. a lesion. The essence of noise is rather that it doesn't "disrupt" the input, but distorts it.
8. Line 548ff: "This is particularly challenging in neural networks with non-linearities, such as rectified linear units (ReLUs). In the specific case of neural networks, this issue has caused extensive debate (28, 29). Here we mitigate this problem by verifying that our results are consistent with two estimators, both discrete and continuous, and with different hyperparameter settings." In general I found your "Limitation and future work" discussion very elucidating, because it showed further reasons for your methodology (like the quote aboved). I would have even liked to have more of this in the Methods section.
9. To make the paper a bit more self-contained I missed a very short description of the DM family tasks, maybe one example of a the non-context task and one of a context task.
10. To produce the results for figure 5e)-left you averaged the synergy over all congruent vs. all incongruent task results, is this correct? I'm just surprised that the probability density functions show such Gaussian-like profiles, even though the tasks are categorically different. Why do you think this is the case?

**References**


Barrett, A. B. (2015). Exploration of synergistic and redundant information sharing in static and dynamical Gaussian systems. *Physical Review E*, *91*(5), 052802. https://doi.org/10.1103/PhysRevE.91.052802

Bowers, J. S. (2017). Parallel Distributed Processing Theory in the Age of Deep Networks. *Trends in Cognitive Sciences*, *21*(12), 950 961. https://doi.org/10.1016/j.tics.2017.09.013

Hinton, G. E., Srivastava, N., Krizhevsky, A., Sutskever, I., & Salakhutdinov, R. R. (2012). *Improving neural networks by preventing co-adaptation of feature detectors* (arXiv:1207.0580). arXiv. http://arxiv.org/abs/1207.0580

Luppi, A. I., Mediano, P. A. M., Rosas, F. E., Holland, N., Fryer, T. D., O Brien, J. T., Rowe, J. B., Menon, D. K., Bor, D., & Stamatakis, E. A. (2022). A synergistic core for human brain evolution and cognition. *Nature Neuroscience*, *25*(6), 771 782. https://doi.org/10.1038/s41593-022-01070-0

Molano-Mazon, M., Barbosa, J., Pastor-Ciurana, J., Fradera, M., Zhang, R.-Y., Forest, J., Del Pozo Lerida, J., Ji-An, L., Cueva, C. J., De La Rocha, J., Narain, D., & Yang, G. R. (2022). *NeuroGym: An open resource for developing and sharing neuroscience tasks* [Preprint]. PsyArXiv. https://doi.org/10.31234/osf.io/aqc9n

Srivastava, N., Hinton, G., Krizhevsky, A., Sutskever, I., & Salakhutdinov, R. (2014). Dropout: A Simple Way to Prevent Neural Networks from Overfitting. *The Journal of Machine Learning Research*, *15*(1), 1929 1958.

Williams, P. L., & Beer, R. D. (2010). *Nonnegative Decomposition of Multivariate Information* (arXiv:1004.2515). arXiv. http://arxiv.org/abs/1004.2515
