# Supplementary material for: Synergistic information supports modality integration and flexible learning in neural networks solving multiple tasks
Source: PLoS Comput Biol. 2024 Jun 3;20(6):e1012178. doi: 10.1371/journal.pcbi.1012178 (PMC11175422; doi:10.1371/journal.pcbi.1012178)
Supplement: Attachment — Submitted filename: PLOSCB_review_response.docx [file pcbi.1012178.s003.docx]

**Responses to comments from reviewers of paper**

**Manuscript ID *52617d2b8b3b527e***

**“Synergistic information supports modality integration and flexible learning in neural networks solving multiple tasks”**

**RC** *Reviewer’s Comment*

**AR** Authors’ Response

We would like to thank the editor and the reviewers for their thoughtful work in reviewing our paper. The received comments have enabled substantial improvement on the revised manuscript. In order to aid the revision process, the modified text in the resubmitted manuscript has been highlighted.

On behalf of all authors,

Alexandra Maria Proca

**Responses to comments from Reviewer 1**

**RC** *This article addresses important aspects of studying artificial and biological neural systems. In particular, it hypothesizes and tests several functions that can be assigned to the individual components of Partial Information Decomposition (PID): it assigns the function of robustness for redundant information, specialization for unique information, and modality interpretation, flexibility as well as efficient coding for synergistic information.*

*I enjoyed reading the article, learned something new (about PID for instance), and acquired certainty for things already suspected (regarding the effects of dropout or the effects of sequential-vs.-interleaved training for different sets of tasks, for instance). I think that the paper is sound, that it represents a significant contribution, and that it is of interest to the cognitive neuroscience community.*

*Since I did not find any major content-related or methodological problems, I only have a list of comments and remarks that might rather reflect my own preferences as a reader than actual weaknesses. I don't expect my points to be met in any specific way, but I think that focusing on some of them could improve the paper if you decide to do so:*

**AR** We thank the reviewer for the positive feedback and for the very helpful suggestions.

**RC** *You address the cognitive neuroscience community, which I find appropriate and promising, as I see the main motivation for PID there (more than, e.g., in the purely theoretical field of explainable AI). The relation to cognitive neuroscience is made explicit in three ways: 1., ANNs are somewhat similar to biological neural networks, therefore---although biologically implausible---they can help to extract the fundamental principles of cognitive systems; 2., the 3rd experiment uses the NeuroGym task suit of Molano-Mazon et al. (2022) which has been used for some experiments in decision-making in cognitive neuroscience; and 3., you frequently relate your findings to the information integration in cortical association areas which is largely based on the Luppi et al. (2022) paper.*

*Since the third relation is mostly conceptual and not exactly an outcome of this study’s own results, I would find it more interesting to discuss the relations of 1. and 2. more extensively. Relation 1. has been discussed extensively under the name of distributed vs. localist processing for example (see Bowers, 2017, e.g. and referencing earlier work in Parallel distributed Processing); and for relation 2. it might be interesting to discuss experimental results that have used the NeuroGym test suite for animal experiments.*

**AR** We thank the reviewer for this suggestion. We have added additional discussions of recent work studying distributed and local processing in ANNs, as well as a brief review of work using the Neurogym suite in ANNs and animals, in the Discussion and Results sections, respectively. For convenience, we copy here the new paragraphs added to the revised version of the manuscript:

“Our study of the relationship between ANNs and biological neural networks has interesting parallels to the study of distributed versus localist processing. In particular, parallel distributed processing (PDP) models from psychology [59-61] claim that the brain’s encoding of information is distributed, rather than represented locally in specialized representations, and non-symbolic. Indeed, the predecessors of modern deep learning models, PDP models have been successful in helping to explain computation underlying cognition. However, theories about localist processing have found support in ANNs which, although designed to be parallel and distributed, also appear to develop semantically meaningful categories within specialized units. Our work further unites these two perspectives, as we observe networks strike a balance between specialization and redundancy versus distributed synergistic representations. We suggest that both localist and distributed forms of information processing may be utilized in different settings, depending on the type of computation being performed, noise, and perhaps other factors such as the environmental statistics related to the information being represented.”

“Previous work has used this framework to compare ANNs to animal studies, for example in studying population gating of task-relevant features in the primary auditory cortex of rats [40], and replicating suboptimal behavior produced by structural priors in rats [41].”

**RC** *Sometimes I thought that the discussions about the failures and the results, especially for the first experiment (e.g. lines 165 to 224), could be made more concise.*

**AR** We thank the reviewer for this suggestion. We have condensed the discussion of the results for the first experiment (lines 165 to 224 in the original submission, lines 212 to 264 in the revised manuscript).

**RC** *The redundancy functions (Barrett, 2015) and (William & Beer, 2010) were previously unknown to me, but since this is where the "juice" of PID is, I expected a little intuition as to what both calculations are and also what their functional difference is (either in the background section or in the methods section next where you present the explicit formulas for calculating the measures).*

**AR** We thank the reviewer for this suggestion. We have added a brief description in the Methods section to provide intuition behind these two PID measures and how they relate to each other on a higher level, copied here for convenience:

“To provide some intuition, $I_{MMI}$ computes redundancy as the minimum amount of information any single source provides about the target and synergy as the minimum amount of information lost about the target by removing any single source from the entire set of sources. $I_{min}$ computes redundancy and synergy similarly, but by instead taking the *expected value* with respect to the target of the minimum amount of information provided by any single source or lost by removing any single source.”

In addition, in response to a comment from Reviewer 2, we have also added a paragraph to the Background section with more context on redundancy functions.

**RC** *With regard to Fig.2 c), I would advise to write that p=0.0 and p=0.5 denote dropout values. It can be deduced from the context (particularly if the image is positioned within the text), but mentioning it in the description as well would make it easier.*

**AR** We thank the reviewer for this suggestion and apologize for the ambiguity. We have now clarified that p=0.0 and p=0.5 refer to dropout values in the caption for Fig. 2.

**RC** *Maybe it is interesting to mention how this dropout interpretation in terms of redundancy compares to the traditional interpretation of Hinton et al. 2012, Srivastava et al. 2014 as preventing complex co-adaptations.*

**AR** We thank the reviewer for this suggestion. We have added a discussion of how the reduction in synergy and increase in redundancy produced by dropout relate to the interpretation of dropout preventing complex co-adaptations. We copy it here for convenience:

“Our interpretation of dropout provides a complementary perspective to the classic view of dropout as preventing complex co-adaptations [46, 47]. Rather than learning complex, distributed ‘co-adaptations,’ which may link correlations in the input between task-relevant and task-irrelevant information, dropout effectively sparsifies the network by increasing the risk of losing information, causing the network to learn to overrepresent task-relevant information (redundancy) while decreasing information coded across many neurons, which has a higher risk of being disrupted (reduced synergy), and to discard task-irrelevant information which may interfere.”

**RC** *Several ArXiv Preprints seem not to be cited correctly, only the first author and the url is mentioned.*

**AR** We apologize for our oversight in citation formatting. This has now been corrected.

**RC** *Line 436ff: "Synergistic information, however, is also more vulnerable to noise (10), because a disruption in a single source could disrupt information synergistically held together with other sources." I am unsure whether I would call the susceptibility to noise, a "disruption", i.e. a lesion. The essence of noise is rather that it doesn't "disrupt" the input, but distorts it.*

**AR** We thank the reviewer for raising this point. We have replaced “disruption” with “distortion” in line 436 of the original submission (line 491 in the revised manuscript).

**RC** *Line 548ff: "This is particularly challenging in neural networks with non-linearities, such as rectified linear units (ReLUs). In the specific case of neural networks, this issue has caused extensive debate (28, 29). Here we mitigate this problem by verifying that our results are consistent with two estimators, both discrete and continuous, and with different hyperparameter settings." In general I found your "Limitation and future work" discussion very elucidating, because it showed further reasons for your methodology (like the quote above). I would have even liked to have more of this in the Methods section.*

**AR** We thank the reviewer for this suggestion. We have incorporated the points raised in the Limitations and future work section in the Methods section as well to further motivate our methodology.

**RC** *To make the paper a bit more self-contained I missed a very short description of the DM family tasks, maybe one example of a non-context task and one of a context task.*

**AR** We thank the reviewer for this suggestion. We have now added a short description of the DM family tasks, copied here for convenience:

“This set of tasks is based on various decision-making tasks used in neuroscience and psychophysics [43-45], involving the presentation of two simultaneous stimuli (e.g., numeric value of the input at a particular dimension) within a specific modality (set of input dimensions; e.g., DM1 presents stimuli in modality 1 and DM2 in modality 2), after which models must indicate which stimulus is stronger (higher in value). In contextual decision-making tasks (CtxDM1, CtxDM2), a second set of stimuli is also presented in the other modality (the remaining set of input dimensions) and must be ignored. In non-contextual tasks (DM1, DM2), stimuli are not presented in the other modality.”

**RC** *To produce the results for figure 5e)-left you averaged the synergy over all congruent vs. all incongruent task results, is this correct? I'm just surprised that the probability density functions show such Gaussian-like profiles, even though the tasks are categorically different. Why do you think this is the case?*

**AR** Yes, this is correct. The congruent tasks consist of DM1&CtxDM1 and DM2&CtxDM2 pairs, which only differ from each other in which one of the two modalities the stimulus appears in; thus, synergy would be expected to be similar in both pairs of tasks. The set of incongruent tasks instead consists of DM1&DM2, DM1&CtxDM2, DM2&CtxDM1, and CtxDM1&CtxDM2. Similar to the congruent case, it would be expected that DM1&CtxDM2 and DM2&CtxDM1 would yield similar levels of synergy because they only differ from each other in terms of which one of the two modalities is presented with/without a ‘distractor’ stimulus (i.e., they are ‘swapped’). Indeed, DM1&DM2, and CtxDM1&CtxDM2 do differ from each other and the other pairs of tasks categorically; whereas the other pairs of incongruent tasks contain one contextual task (with distractor stimuli in the modality that must be ignored) and one non-contextual task (with no distractor stimuli), either both (CtxDM1&CtxDM2) or neither (DM1&DM2) task are contextual. However, it is likely that networks trained with interleaving on these task pairs show similar synergistic profiles to the other incongruent pairs of tasks because they learn to attend to one modality in one context and attend to the other in the other context in order to successfully perform the task, regardless of whether or not there is the presence of a distractor in the modality that must be ignored. Thus, from a functional perspective, networks trained on these different tasks may be exhibiting similar behaviors/strategies, despite being trained on tasks that are categorically different, resulting in similar behavior in synergy.

**Responses to comments from Reviewer 2**

**RC** *Proca et al. employ a recent extension of information theory, called partial information decomposition to the analysis of distributed computation in neural systems learning multiple tasks. They show that the mutual information between subsequent processing stages (or inputs and processing stages) moves into PID-atoms of higher synergy when networks learn multiple diverse tasks. They conclude that synergy in these networks enables flexible (and efficient) learning. While the efficiency gain via synergistic modes of operation may have been expected based on purely theoretical considerations about the size of possible coding spaces, the flexibility is indeed a novel and important result.*

*This is a timely and important study that highlights the shortcomings of classic information quantities in the analysis of distributed computation and shows how to overcome these shortcomings via partial information decomposition. The example cases presented are well chosen and of high relevance, and the simulations and analysis are performed thoroughly. As such I think this manuscript presents an important advance in the field. I would like to stress this aspect here, as I will list a range of criticisms below that need to be addressed, and may sound harsh, but which also can be addressed and should in my opinion not stand in the way of the ultimate success of this study.*

*Since it is one of the first studies of its kind, and therefore lays a foundation for future investigations of this kind, is has to be held to the higher standards than usual in order to avoid that the field of PID analyses of distributed computation drifts into some sort of ad-hoc, hand waving ‘reading of tea-leaves’ as has for example happened with the now infamous cross-frequency coupling analyses of the past decade.*

*To this end I think it is mandatory that the following issues be thoroughly addressed:*

**AR** We thank the reviewer for the positive feedback and the very helpful comments.

**RC** *I think it is not OK to totally gloss over the problem of various PID measures in the Background section on partial information decomposition, and to defer any further discussion to the appendix/supplementary. This is because to date >25 different PID measures exist, with many of them shown to solve highly specific PID problems. The problem solved by a particular PID is tightly related to what is called the operational interpretation of the respective PID, i.e. its very meaning. For example, the popular measures I_BROJA and I_CCS have thorough decision or game theoretic operational interpretations, respectively, but using I_BROJA in a game theoretic setting would be just as wrong as using I_CCS in the decision theoretic setting, as has been demonstrated explicitly. Thus, the application of a specific PID measure to a neuroscience or machine learning question has to be justified thoroughly by the corresponding operational interpretation of the measure.*

*It is also well known that various PID measures can strongly disagree in certain problem settings [1], and that this must be this way, simply because their measurements have entirely different interpretations. So I would like to ask the authors to make explicit the link between the operational interpretation of the two PID measures they used in their study and the problem setting addressed in the manuscript.*

**AR** We thank the reviewer for raising this important issue. Although we do not know of any meaningful operational definitions for the two PID measures we use, we have included a discussion in the Background section highlighting the existence of many PID measures with different behaviors and operational definitions, and our reasons for our specific choices. We copy the new paragraph here for convenience:

“Although PID proposes the distinction of unique, redundant, and synergistic information, it does not prescribe a unique method for computing these measures and to date there is no single widely-accepted method of doing so. Consequently, many different PID measures have been formulated which capture different aspects of multivariate information, some which have been shown to provide a specific operational interpretation (e.g. the payoff in a suitably defined game [28] or the compression through a suitably defined channel [29]). Although past work has shown that in practice different PID measures tend to yield similar results [7, 29], it has also been shown that there are situations where different PID can strongly disagree [30]. Taking these considerations into account, we compute all measures using two different redundancy functions, $I_{min}$ [6] and $I_{MMI}$ [31], and a range of estimator parameters, to ensure our results are consistent. Although these do not have a meaningful operational interpretation, there are two reasons that make them very suitable for our study: 1) they have previously been used to study neural data which we aim to explain using artificial neural networks; and 2) unlike most other measures, $I_{min}$ and $I_{MMI}$ are more pragmatically useful, since they allow us to efficiently estimate synergy in systems with potentially many sources without computing the full PID lattice (mathematically, in both cases the inclusion-exclusion principle can be applied to yield a measure of “union information” in closed form). For additional discussion on the field of PID measures, we point the reader to the review of [32].”

**RC** *The issue of binning and the problems related to binning choices should be treated better. It is obvious from the simple observation that in the limit of very few bins PIDs tend to turn out too high levels of redundancy, while in the limit of too many bins and estimation from finite data will be almost certainly a one-to-one mapping of each specific pair of inputs to one specific output for that pair, and thus, just synergy. This seems to prohibit the use of binned variables in a PID setting – unless the binning can be motivated extrinsically, i.e. in the sense that the computational system itself uses that binning or has a sensitivity reflecting that binning. While I accept the difficulty that so far only very few PID measures and estimators for systems of mixed discrete and continuous variables exist (e.g. [4,5], but there is no code available at present), this problem cannot simply be ignored. A more acceptable way to tackle the problem of mixed discrete-continuous observation spaces would perhaps be to use a small amount of Gaussian noise on the discrete parts of the observation space and the Gaussian copula method instead of some ad-hoc binning; a less desirable alternative approach would be to present results over a wide range of bin numbers or widths and to thoroughly discuss the binning issue. I think this point is very important so as not to set a bad precedent that is then in the future cited in order to justify the practice of binning in the PID setting, as this would certainly damage the field.*

**AR** We thank the reviewer for raising this important and nuanced issue. We fully agree with the reviewer that binning-based estimators of information quantities are generally suboptimal, but we are inclined to disagree with the conclusion that these limitations outright prohibit the use of binned variables in PID. On one extreme, it is clear that there are binary systems without redundancy (e.g. the XOR gates), so even systems with very few bins can have no redundancy. On the other extreme, there are some PID measures (including MMI) that converge to their continuous counterparts under very thin binning, so a tendency towards synergy may be more an artifact of finite data rather than a fundamental limitation of binning for PID. We strongly agree with the reviewer that the estimation of PID quantities is a greatly understudied problem, and all these considerations are far from obvious.

We have taken two steps to alleviate the reviewer’s concerns about our estimator. First, we have followed the reviewer’s suggestion of repeating our analyses over a range of bin numbers to confirm our results are qualitatively consistent. The results of this validation were completely in agreement with our conclusions, and are shown in the Supporting Information (S1 Table). Second, we have added a detailed discussion in the Methods section about the estimation of PID quantities and why this is particularly challenging in the study of neural networks. This discussion is copied here for convenience:

**“Discretization of Activations**

Discretization of continuous values, such as the activations of neural networks, presents a challenge for accurately approximating probability distributions, as the choices of bin width and range are likely to influence resulting measures. To address potential issues with binning selection, in addition to the discretization method we employ specific to each experimental setting (logic gate and Animal-AI, specified in the methods section of the corresponding setting), we also compute PID measures using 4 other binning strategies and compare them to summary statistics of our main results (S1 Table), finding the results to be qualitatively similar.

In particular, for each layer and each model seed, we select the maximum of values to be binned across as the 3rd quartile + 1.5$\times$IQR of the set of sampled activations, where IQR is the interquartile range. The minimum is selected as the maximum between the 1st quartile - 1.5$\times$IQR and 0, since our ReLU activations constrain all samples to be nonnegative. We then take an even-frequency split of the samples between the resulting range according to the number of bins (e.g., for 2 bins splitting by the median, for 4 bins splitting by quartiles, etc.). We employ this method across 3, 4, 5, and 10 bins. In the Animal-AI experiments, we discretize the continuous parts of the input with the same number of bins used for the layer discretization based on the IQR, but with evenly spaced bins across the entire observation space.”

**RC** *The references to more advanced measures seems to be lacking important recent developments, both, for multivariate PIDs (e.g. [2]), PIDs for continuous variables (e.g. [3]) and the combination of these properties (e.g. [4,5]) – just to name a few. In this context it may also be in order to mention that some of these multivariate measures indeed have been applied to deep neural networks – e.g. up to five-input PIDs [7].*

**AR** We thank the reviewer for this suggestion. We have added more references to reflect more recent developments.

**RC** *A pointer to the overall structure of the field of PID measures would be in order, to give the reader a feeling for the possibilities and limitations of the concept. The introductory review [6] seems to be a frequently cited reference to consider here but any other recent review on the overall structure of the field would be just as fine.*

**AR** We thank the reviewer for this suggestion. We have given an explicit pointer to this review in our discussion of PID measures in the Background section and added it to our references.

**RC** *The use of PID has rapidly gained momentum in the machine learning world. It may be good to stress the importance of the method applied in the present study and thus the validity of the presented approach to highlight this general interest and acceptance of PID in the machine learning world by including some references documenting this. One of the reasons for why this may be beneficial for the readers of the current manuscript may be that they can then find the approach that best fits their particular use case in terms of operational interpretations – as explained above.*

**AR** We thank the reviewer for this valuable suggestion to improve the impact of our work within the machine learning community. We have added a paragraph early in the Introduction to motivate the use of PID in the context of machine learning. We copy the paragraph here for convenience:

“In addition to its application to neuroscience, PID is also rapidly gaining traction in the field of machine learning. While the information bottleneck theory is one of the most well-known applications of information theory to neural networks [13-16], the studies used Shannon mutual information which does not capture additional interactions between variables. PID now offers an elegant framework for studying distributed information processing and interactions between parts of a system, which becomes increasingly important as the field moves towards further developing theory of deep learning, better interpreting existing models, and designing new architectures and algorithms that may employ particular information processing strategies. Furthermore, this approach offers the possibility of understanding of neuronal cooperation and organization in networks that are often difficult to interpret. Recent work developed a differentiable PID measure [17, 18], which has been used to specify local learning rules optimizing for interpretable information-processing goals in neural networks [19]. Other studies have used PID for feature selection [20], analyzing training dynamics in convolutional neural networks [21], estimating redundancy in factor graphs [22], and studying representational complexity in ANNs [23], among other work [24, 25].”

**RC** *Unfortunately it remains unclear to me how the full order PID decomposition was performed. The authors write: “ All calculations are performed over sets of sources of size K, where K is either the cardinality of the full set of sources (either the whole layer or the whole input space; referred to as full-order); or K = 2 (2nd-order)…” When K is the cardinality of the full set of sources, then there is only one such set (containing all source variables), and we thus seem to obtain a joint mutual information – is this what was done? If not please rephrase and include a formula for the full-order decomposition, and the maximum number of atoms resulting from the cardinality used in the experiments. To restate my problem: I do understand the 2nd-order decomposition, but not the full-order counterpart.*

**AR** We apologize for the ambiguity in our phrasing. Yes, our definition of full-order decomposition refers to K being equal to the cardinality of the full set of sources, such that there is only one such set (containing all source variables) and the decomposition is taken over the joint mutual information of all source variables. We have added the following sentence and formula to the Methods to further clarify this:

“More specifically, for a set of sources $\boldsymbol{X}=\{X_{1},X_{2},\ldots,X_{M}\}$ and target $Y$, the *K*-order redundancy $R^{(K)}$ is defined as the average of the redundancy of subsets of sources of cardinality *K*:

$R^{(K)}(\boldsymbol{X};Y)=\langle R(\boldsymbol{A};Y)\rangle_{A}$, with $\boldsymbol{A}\subseteq\boldsymbol{X}, \left| \boldsymbol{A} \right|=K$

In the case of 2^nd^-order, $K=2$ and in full-order, $K=M$ (therefore there is only one set being considered in the full-order case). In other words, for the case of full-order decomposition, there is only one set containing all source variables and the decomposition is taken over the joint mutual information of all source variables.”

*References:*

*[1] Kay, J. W., Schulz, J. M., & Phillips, W. A. (2022). A comparison of partial information decompositions using data from real and simulated layer 5b pyramidal cells. Entropy, 24(8), 1021.*

*[2] Chicharro, D., & Panzeri, S. (2017). Synergy and redundancy in dual decompositions of mutual information gain and information loss. Entropy, 19(2), 71.*

*[3] Pakman, A., Nejatbakhsh, A., Gilboa, D., Makkeh, A., Mazzucato, L., Wibral, M., & Schneidman, E. (2021). Estimating the unique information of continuous variables. Advances in neural information processing systems, 34, 20295-20307.*

*[4] Schick-Poland, K., Makkeh, A., Gutknecht, A. J., Wollstadt, P., Sturm, A., & Wibral, M. (2021). A partial information decomposition for discrete and continuous variables. arXiv preprint arXiv:2106.12393.*

*[5] Ehrlich, D. A., Schick-Poland, K., Makkeh, A., Lanfermann, F., Wollstadt, P., & Wibral, M. (2023). Partial Information Decomposition for Continuous Variables based on Shared Exclusions: Analytical Formulation and Estimation. arXiv preprint arXiv:2311.06373.*

*[6] Gutknecht, A. J., Wibral, M., & Makkeh, A. (2021). Bits and pieces: Understanding information decomposition from part-whole relationships and formal logic. Proceedings of the Royal Society A, 477(2251), 20210110.*

*[7] Ehrlich, D. A., Schneider, A. C., Priesemann, V., Wibral, M., & Makkeh, A. (2023). A measure of the complexity of neural representations based on partial information decomposition. Transactions on Machine Learning Research, 5.*

**Responses to comments from Reviewer 3**

**RC** *The paper presents a really nice analysis of information encoding in neural network models that learn tasks. The main result is the emergence of more and more synergistic neurons as the networks learn to perform more and more refined tasks. The analyses are well done and the conclusions well-reasoned and well-taken. I only have a few requests for clarification.*

**AR** We thank the reviewer for the positive feedback and for the very helpful suggestions.

**RC** *The authors cite extensively their own work and that of others about synergy in brain data. However, most of the cited literature is describing analyses of aggregate neural signals, such as fMRI that have relatively little to reveal about computations at the population or single neuron levels. Surprisingly, the authors do not consider instead the coding of information with single neuron resolution, which has been studied as well (e.g. Sherrill et al (2021) PLoS Comput Biol 17(7):e1009196. https:// doi. org/ 10. 1371/ journ al. pcbi. 10091 96; Valente et al (2021) Nat Neurosci 24(7):975–986. https:// doi. org/ 10. 1038/ s41593- 021- 00845-1;*

*Koçillari et al. Brain Informatics (2023) 10:34 https://doi.org/10.1186/s40708-023-00212-9) These single neurons resolution studies would be more relevant and directly comparable to the one presented here in the analysis of artificial neural networks, and some of these single neuron papers use exactly the same methods (information decompositions) used by the authors for the analysis of neural networks. Importantly, some of these papers have found synergy and recognized its important function. However, all the single neuron papers have also found redundancy and reported convincing evidence of its utility at the neural network computation level. The authors should make more contact with the synergy/redundancy single neuron literature and also discuss more in Discussion the relative merits of synergy and redundancy for neural computations.*

**AR** We thank the reviewer for raising this point, and apologize for the omission. While our initial motivation was to better understand the results yielded by studies using fMRI data, we agree that computations at the single neuron and population level are very relevant in the context of PID. We have now made an effort to make more explicit links to these points and the relevant literature. We have added a new paragraph to the Discussion, and we show it here as well for convenience:

“In addition to studies featuring information-decomposition analyses of aggregate neural signals, such as fMRI, which initially motivated the current work, other work has used PID to analyze computations at the population and single neuron levels, which offer a more natural analogue to ANNs. One study [56] found that in *in vitro* spiking cortical neurons of mouse somatosensory cortex, greater recurrent information flow (in terms of number of recurrent edges and strength of connectivity) was associated with higher synergy, whereas greater feedback relative to feedforward information was found to result in less synergy. In alignment with the authors’ discussion, our study suggests that this may be explained by the importance of recurrent connections for lateral integration of multiple features, which elicits increased synergy, whereas feedback may reduce the variance of feedforward information and its corresponding strength, perhaps performing less integration of distributed sources of information and instead specializing along certain pathways. While other studies have recognized the utility of synergistic processing, many have also highlighted the important role of redundancy in neural computation. For example, noise correlations in the association cortex of mice were shown to predict better task performance despite decreasing sensory information [57]. One explanation offered was that such correlations are the result of redundancy in neural representations used to enhance signal propagation. Although these results seemingly conflict with other evidence of association cortices being highly synergistic, the particular study used Pearson correlation and PCA, both of which cannot reveal the separate contributions of unique, redundant, and synergistic effects, and thus it is difficult to evaluate exactly how these findings translate to the framework of PID specifically. Nevertheless, other work has indeed revealed that increased redundancy is associated with correct behavioral responses related to perceptual discrimination in the mouse auditory cortex [58]. This aligns with our finding that redundancy offers robustness in the presence of noise, and our prediction that such robustness is especially realized in low-level sensory cortices.”

**RC** *In lines 62, the authors write “Synergy and related quantities, such as integrated information (8),”. I would disagree with this statement. Integrated information cannot be reduced to synergy. There are analogies and differences, but I believe that the statement as written is not correct. I invite the authors to rewrite it in a more precise way.*

**AR** We apologize for the imprecise phrasing of this sentence. We have rewritten the sentence as, “Synergy and integrated information, of which synergy is a constituent component, have also been found to dominate in …” – which is a more precise statement of the findings in Ref. [8].
